# Characterization of a Putative New Member of the Genus *Potyvirus* from Kudzu (*Pueraria montana* var. *lobata*) in Mississippi

**DOI:** 10.3390/v15112145

**Published:** 2023-10-25

**Authors:** Nina Aboughanem-Sabanadzovic, Ronald Christian Stephenson, Thomas W. Allen, Alan Henn, William F. Moore, Amanda Lawrence, Sead Sabanadzovic

**Affiliations:** 1 Institute for Genomics, Biocomputing and Biotechnology, Mississippi State University, Mississippi State, MS 39762, USA; nja62@msstate.edu; 2 Department of Biochemistry, Molecular Biology, Entomology and Plant Pathology, Mississippi State University, Mississippi State, MS 39762, USA; rstephenson9@unl.edu (R.C.S.); ahenn@plantpath.msstate.edu (A.H.);; 3 Delta Research and Extension Center, Mississippi State University, Stoneville, MS 38776, USA; tallen@drec.msstate.edu; 4 Institute for Imaging and Analytical Technologies, Mississippi State University, Mississippi State, MS 39762, USA; alawrence@i2at.msstate.edu

**Keywords:** kudzu, symptom, virus, potyvirus, high-throughput sequencing, aphid transmission, soybean, virus taxonomy

## Abstract

Kudzu (*Pueraria montana* var. *lobata*), a plant native to Southeastern Asia, has become a major noxious weed covering millions of hectares in the Southern United States. A kudzu patch displaying virus-like symptoms located in Ackerman, northeastern Mississippi (MS), was used as a source for virus isolation and characterization involving mechanical and vector transmission, ultrastructural observation, surveys, Sanger and high-throughput genome sequencing, and sequence analyses. The results revealed the presence of a new potyvirus in infected kudzu, closely related to wisteria vein mosaic virus (WVMV) and provisionally named kudzu chlorotic ring blotch virus (KudCRBV). Genome features and pairwise comparison with six WVMV genomes currently available in GenBank and three additional isolates from MS sequenced in this work suggest that KudCRBV is likely a member of a new species in the genus *Potyvirus*. Furthermore, under experimental conditions, KudCRBV was successfully transmitted by cotton and potato aphids and mechanically to soybean and beans. A state-wide survey revealed several kudzu patches infected by the virus in northern MS.

## 1. Introduction

Kudzu (*Pueraria montana* var. *lobata* (Willd) Maesen & Almeida) is a leguminous trailing/climbing perennial vine originating from Southeastern Asia. It was originally introduced to the United States during the Philadelphia Centennial Exposition in 1876 and was publicized as an easy-growing ornamental plant. Shortly afterwards, kudzu was presented at the New Orleans exposition in 1883, attracting the interest of homeowners as an ornamental plant for use in residential ornamentation and for shading of porches [1]. Kudzu’s prominence grew additionally in the early years of the 20th century, reaching its peak between the 1930s and 1950s, due to a promotional campaign of the Soil Conservation Service (also known as the SCS, which is now known as the National Resources Conservation Service or NRCS) promoting this plant as a useful tool in reducing and controlling soil erosion problems. During that period, 85 million kudzu plants were freely distributed to landowners and planted in the Southeastern U.S. In addition, government agencies organized systemic planting throughout that region.

**Figure 1:**
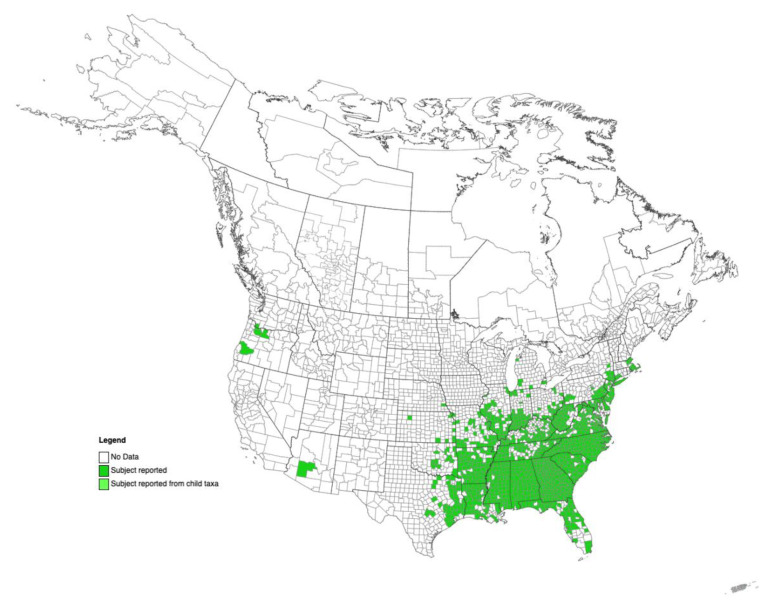
Distribution map of kudzu (*Pueraria montana* var. *lobata*) in the U.S. Map retrieved from EDDmaps online resource (https://www.eddmaps.org/distribution/uscounty.cfm?sub=2425; accessed on 29 August 2023).

Kudzu adapted exceptionally well to the climate of the Southern U.S. As a result, it continued to expand quickly, spreading to 31 states, and became a serious and costly problem. Current estimates suggest that kudzu approximately covers more than 3 million ha in the Southeastern U.S. and continues to expand at an approximate rate of 50,000 ha/year [1,2]. Due to its extremely rapid spread and negative impact on the ecosystem, kudzu gained the nickname “the vine that ate the South”, as it is most established in that region of the U.S. Nevertheless, according to data presented in EDDMapS—the Early Detection and Distribution Mapping System (https://www.eddmaps.org/; accessed on 29 August 2023)—kudzu can currently be found growing in Arizona, Oregon, and Washington too (Figure 1), as well as in Canada (https://www.theglobeandmail.com/news/national/vine-that-ate-the-south-has-landed-in-the-great-white-north/article4287178/; accessed on 30 August 2023).

**Figure 2:**
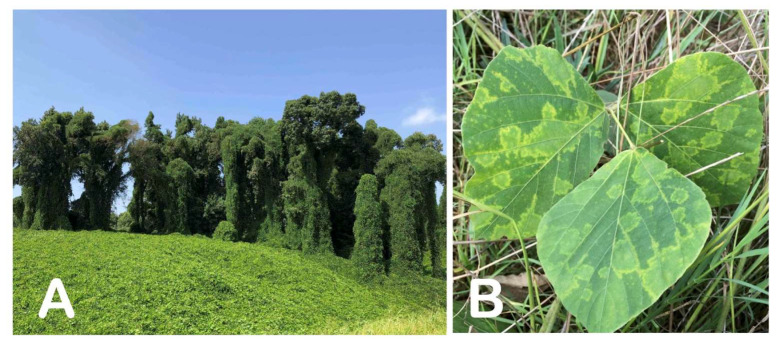
A kudzu patch dominating the landscape along a highway in north Mississippi (**A**) and systemic chlorotic ring blotch symptoms observed on a trifoliate of kudzu sample “Ack01” (**B**) used as a source for the virus characterization.

Since kudzu has an aggressive and rapid growth pattern, it has the ability to outcompete vegetation nearby, from native grasses to fully mature trees, by covering them (Figure 2A) and interfering with their photosynthetic ability, ultimately leading to the loss of native plants and their associated micro- and macroorganisms. Besides its negative environmental and ecological impact (i.e., species extinction, reduction in overall biodiversity, etc.), kudzu causes significant economic damage. The negative economic impact of kudzu is estimated at a USD 100–500 million loss in forest productivity [2,3]. Furthermore, the costs of kudzu control by electrical companies are estimated at approximately USD 1.5 million annually. A recent Monte Carlo simulation study based on currently infested locations in Oklahoma was conducted to predict the natural and anthropogenic spread rates over the next five years and to estimate the economic impact on the local forest industry. The results suggested that, over the course of the five-year period, the total timber industry output could be reduced by approximately USD 170 million, impacting almost 800 jobs and highlighting the negative economic impact of further spread of this invasive plant [4].

Kudzu is a host for several important plant pathogens and pests in the U.S. In addition to acting as an additional over-wintering host for the soybean rust fungus (*Phakopsora pachyrhizi*) [5] and serving as a reservoir for the kudzu bug (*Megacopta cribaria*), kudzu is also susceptible to a few viruses. Indeed, infections by tobacco ringspot virus (TRSV, a nepovirus) and soybean vein necrosis virus (SVNV, an orthotospovirus) have been reported from the Southern U.S. over the past decade [6,7,8]. Curiously, until very recently, the only report regarding viruses affecting kudzu from Southeastern and Eastern Asia originated from Vietnam and concerned kudzu mosaic virus (KuMV, a begomovirus) [9]. However, research conducted by Liang and collaborators revealed mixed infections by three viruses in a batch of symptomatic samples collected in Beibei (Chongqing, China) [10]. Two of the viruses detected were determined to be novel viruses in the genera *Crinivirus* (family: *Closteroviridae*) and *Emaravirus* (family: *Fimoviridae*) and were observed in coinfections with KuMV.

In the current paper, we report a comprehensive characterization of a new virus with features typical of potyviruses and which is possibly a member of a new species. Members of the genus *Potyvirus* (family: *Potyviridae*, order: *Patatavirales*) are viruses with flexuous filamentous virions (~700–900 nm long and 11–13 nm wide) containing a genome made up of polyadenylated, positive-sense RNA generally 9.7–11 kb in size [11]. They are transmitted in nature by aphids in a non-persistent manner, and some can also be seedborne. Potyviruses are one of the most numerous groups of plant viruses, and they are currently classified in 201 distinct species following specific demarcation criteria [11,12].

## 2. Materials and Methods

### 2.1. Plant Materials

The primary plant material used for this research was derived from a kudzu plant with the lab code “Ack01”, located in the town of Ackerman, Choctaw County, Mississippi. The plant material displayed virus-like symptoms in the form of chlorotic ring blotches and distinct line patterns (Figure 2B). Additional plant material was collected from 103 distinct kudzu patches throughout Mississippi, and 39 local wisteria (*Wisteria sinensis*) samples were used in various phases of the study (more details provided later in this section).

### 2.2. Double-Stranded RNA Analysis

Double-stranded RNAs (dsRNAs) were isolated from 10 g of foliar tissue collected from the symptomatic sample “Ack01” using double phenol–chloroform extraction followed by CF-11 column chromatography [13]. Foliar tissue from a symptomless kudzu and grapevine leafroll-associated virus 2 (GLRaV-2)-infected *Nicotiana benthamiana* were used as controls. The extracts were submitted to selective enzymatic digestions with RNAse-free DNase, DNAse-free RNAse in high-salt conditions (2X SSC: 300 mM sodium chloride and 30 mM sodium citrate, pH 7.0), and Proteinase K prior to elimination of DNA and ssRNA traces. Purified dsRNAs were analyzed in 1% TAE agarose and visualized by staining with ethidium bromide. The approximate size of the dsRNA under investigation was estimated by comparison with replicative forms of isolates of grapevine leafroll-associated virus 2 [14], bell pepper endornavirus isolate [15], and Japanese holly fern mottle virus isolate DI [16], routinely used as references in our lab at Mississippi State University.

### 2.3. Electron Microscopy

Small foliar tissue fragments, excised from the symptomatic foliage of “Ack01”, were used for observation of possible cytopathological alterations associated with the infection by the studied virus. Fragments were processed according to standard procedures [17], i.e., fixation in 4% glutaraldehyde in 0.05 M phosphate buffer, post-fixation in 1% osmium tetroxide for 2 h at 4 °C, and staining overnight in 2% aqueous uranyl acetate, also at 4 °C. Embedding was in Spurr’s resin after dehydration in graded ethanol. Thin sections were stained with lead citrate before viewing with a JEOL JEM-1400 120 kV transmission electron microscope. Leaf fragments from symptomless kudzu seedling accession no. 9227 (see Section 2.6 for more details) were used as negative controls.

### 2.4. Genome Sequencing, Sequence Assembly, and Analyses

In this study, genome sequencing was performed for the kudzu virus as well as for three isolates of wisteria vein mosaic virus (WVMV) from Mississippi. Sequencing of the kudzu virus was originally performed via the Sanger methodology of random primer-generated cDNA clones starting from a dsRNA template as previously described [18,19].

Genome sequencing of the three WVMV isolates and verification of the kudzu virus genome were performed via Illumina-based high-throughput sequencing (2 × 150 nt pair-end reads) of ribo-depleted total RNA preparations performed on an Illumina HiSeq3000 platform on a custom basis at the Roy J Carver Biotechnology Center (University of Illinois at Urbana–Champaign). For that purpose, total RNA was extracted from 0.1 g of foliar tissue with a Spectrum TM Plant Total RNA kit (Sigma-Aldrich, St. Louis, MO, USA), checked for quality/quantity with a Qubit 3.0 fluorometer (Invitrogen, Waltham, MA, USA), and shipped overnight on dry ice to the sequencing facility. The raw sequencing data (a total of ~35–50 million reads per sample) were initially checked for the quality, and high-quality reads were de novo assembled into larger contigs and further analyzed with the Geneious Prime v. 2022.0.1 software suite (Biomatters, Auckland, New Zealand) and/or the OmicsBox v. 2.2.4 package (BioBam Bioinformatics, Valencia, Spain).

Sequences of the 5′ non-coding regions (NCRs) of virus genomes were completed using a SMARTer RACE 5′/3′kit (Takara Bio/Clontech). The identities of the virus sequences were determined by BLAST comparison (BLASTn and BLASTx) against the GenBank database in June/July 2022. A subset of WVMV complete genomes available on 1 December 2022 was downloaded to a local database and used for in-depth pairwise comparisons with the studied virus. Pairwise genetic identity calculations were estimated and color-coded distance matrices were generated by the Sequence Demarcation Tool (SDT) [20].

Complete sequences of a kudzu virus were aligned with a set of potyviral genomes representative of the current taxonomy [12] using MAFFT v. 7.407 [21] curated by TrimAl v 1.41 [22]. The best-fit substitution model for the dataset was determined to be LG + F + I + G4 by ModelFinder [23] according to the Bayesian Information Criterion. The maximum-likelihood phylogeny was inferred by IQ-Tree v. 1.16.12 [24] with ultrafast bootstrapping performed by UFBoot2 [25] and visualized with iTOL v5 [26].

### 2.5. State-Wide Survey of Kudzu Patches and Search for Alternative Hosts

To better understand the incidence and distribution of this virus, we performed a survey with sampling throughout the entire state of Mississippi. A total of 103 samples, consisting of 30–35 cm long tips of the vines with young leaves, were collected over the two seasons (late May—early November 2018) from different locations and tested initially in DAS-ELISA using a “general potyvirus” monoclonal antibody kit (Agdia Inc., Elkhart, IN, USA; cat. no. SRA 27200/0500). The samples that were determined to be virus-positive in DAS-ELISA were further tested in RT-PCR with the virus-specific primers (KudV-F 5′CTAAGCCAACTCTGAGACAAATCA3′ and KudV-R 5′GCGGTGGGCCCATGCCCA3′) designed to amplify a 349 nt long portion of the viral genome. The final identification was achieved by custom-based amplicon Sanger sequencing performed at the Eurofins-Operon facility in Louisville, KY, USA.

An investigation into possible alternative hosts of the virus was carried out by collecting and testing tissue from several plants naturally growing in the same ecosystem (Ackerman, MS, USA) as the originally infected kudzu plant. To that purpose, we surveyed the area surrounding the plant “Ack01” for two consecutive years (2019–2020), with sampling performed in May and October of each year. During the survey period, a total of 66 plants belonging to nine different botanical species (Supplementary Table S1) were tested using DAS-ELISA, as described above. Finally, we also tested a total of 85 soybean plants collected from randomly chosen production fields located in the general vicinity of the original source of the virus under study (up to an approximately 5 km distance).

### 2.6. Identification of Possible Vector and Transmission Assays

Two species of aphids collected in Mississippi, identified as the potato aphid, *Macrosiphum euphorbiae* (Thomas), and the cotton aphid, *Aphis gossypii* (Glover), were used in vector transmission trials. The potato aphids were collected from wild lettuce, *Lactuca virosa* L., in the area surrounding the original symptomatic kudzu plant, while cotton aphids were collected from bean *(Phaseolus vulgaris* L.) and cotton (*Gossypium hirsutum* L.) located a few km from the kudzu patch. Transmission experiments were carried out in 2017. Aphids were identified using dichotomous keys available in a reference book on aphids that impact crop plants [27].

Symptomatic kudzu leaf tissue from a “donor” plant Ack01 was placed in a Petri dish and aphids were transferred from natural hosts onto symptomatic tissue using a soft-bristled paint brush and left for two hours. During this period, the aphids were under intermittent observation to ensure that feeding occurred. After two hours, the aphids were transferred to caged kudzu plants with the accession nos. 9227 and 434246 grown using seed obtained from the National Seed Storage Laboratory and soybean (*Glycine max*) plants of the cultivar Asgrow 4903 (AG4903).

To prepare young “recipient” plants for these experiments, kudzu seed were treated as described [28]. Briefly, kudzu seed were soaked in concentrated sulfuric acid for 2 h, rinsed with tap water for approximately 5 min, placed on a wet filter paper in Petri dishes and incubated at 30 °C to promote germination. Following germination, seedlings were transplanted into pots containing sterile soil mix and placed in growth chambers at 24 °C for four weeks, followed by transfer to a greenhouse for an additional month.

A total of 10 kudzu plants (5 plants per accession) were used in this study for each of the two aphid species (a total of 20 plants/experiment). A minimum of ten aphids were carefully placed directly on each individually caged recipient plant. The plants were briefly removed from cages after 24 h, sprayed with an insecticide to kill aphids, and maintained under controlled conditions with daily visual monitoring for symptom development. Soybean cultivar AG4903 was treated in the same manner.

Mechanical transmission to a range of young herbaceous plants was attempted by rubbing sap from symptomatic apical leaves collected from “Ack01” in 0.1 M phosphate buffer, pH 7.2 (1:10 *v*/*w*), onto cellite-dusted leaves of soybean cv AG4903, tobacco (*Nicotiana tabacum* cv ‘Turkish’), bean (*Phaseolus vulgaris* cv ‘Bountiful’), squash (*Cucurbita pepo* cv ‘Zucchini grey’), two cultivars of cucumber (*Cucumis sativus* cvs ‘Hale’s best’ and ‘Sweet delight’), watermelon (*Citrullus lanatus* cvs ‘Crimson sweet’ and ‘Black diamond’), pea (*Pisum sativum* cvs ‘Zipper cream’ and ‘Pinkeye purplehull’), and quinoa (*Chenopodium quinoa*). Each type of plant was represented in 5 replications, except soybean, which was represented by 10 plants. The plants were maintained under a constant temperature regime (approximately 20–22 °C) in a greenhouse for 30 days post-inoculation (d.p.i). The plants were visually observed for symptom expression on a daily basis, starting from three days post-inoculation.

In both transmission experiments, plant material consisting of newly emerged young leaves was harvested at 30 d.p.i and tested individually in DAS-ELISA, followed by confirmatory virus-specific RT-PCR tests, as described in Section 2.6.

## 3. Results

### 3.1. dsRNA Analysis and Electron Microscopy

**Figure 3:**
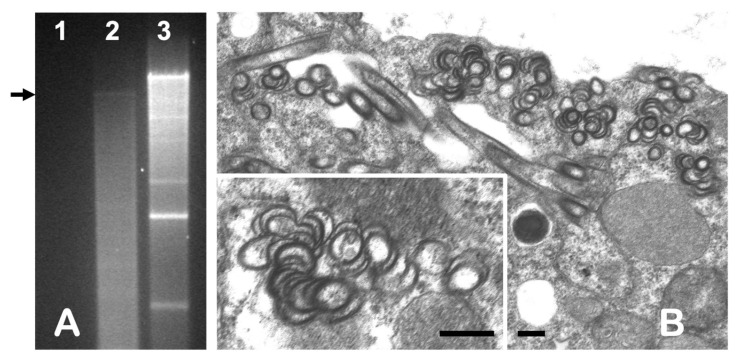
(**A**) Agarose gel electrophoresis of dsRNAs extracted from infected kudzu “Ack01” (lane 2, arrow) compared to extracts from symptomless kudzu (lane 1) and grapevine leafroll-associated virus 2 (GLRaV2)-infected *Nicotiana benthamiana* (lane 3), used as controls. (**B**) Abundance of pinwheels and laminar inclusions, indicative of potyvirus infections, observed in tissue collected from symptomatic kudzu. An enlarged view of a pinwheel structure (inset). Bars represent 100 nm.

Analysis of dsRNA in agarose gel electrophoresis revealed the presence of a single faint band in extracts from the symptomatic kudzu “Ack01”, indicating the presence of the virus, while no visible bands were identified in symptomless kudzu plants processed during the same experiments and used as a control (Figure 3A). The size of a band was estimated at approximately 10 kbp by comparison of its electrophoretic mobility with several dsRNA standards available in our lab.

Electron microscope observation of thin-sectioned tissue from “Ack01” revealed cells containing pinwheel inclusions, scrolls, and laminar aggregates typical of those associated with infection by a potyvirus (Figure 3B). Such inclusions were absent in the controls. Positive results in DAS-ELISA experiments against the “universal” potyvirus monoclonal antibody confirmed the potyviral nature of the virus under study, for which, based upon the symptoms observed on the original host, we propose the name kudzu chlorotic ring blotch virus (KudCRBV).

### 3.2. Genome Sequences of a Virus from Kudzu and Their Analyses

**Figure 4:**
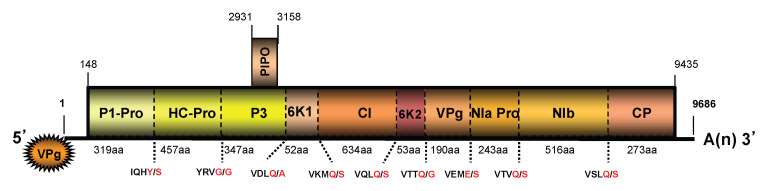
Diagrammatic representation of the genome organization of kudzu chlorotic ring blotch virus isolate Ack01 (KudCRBV-Ack01), with main nt coordinates indicating positions of the two ORFs. The organization is typical of members of the genus *Potyvirus*. The length of putative mature proteins along with predicted cleavage sites are indicated below. Boxes indicate ORFs, horizontal lines indicate non-coding regions, dashed vertical lines indicate presumed cleavage sites along with the amino acid sequences. Mature products encoded by the large ORF are also indicated.

The complete KudCRBV genome was determined to be 9686 nt in size, excluding a polyA tail, containing two open reading frames (ORFs; Figure 4). The genome sequence starts with a nonanucleotide “5′AAAUUAAAA” similar to the initial nucleotide motifs in several potyviruses (i.e., watermelon mosaic virus, WMV, and soybean mosaic virus, SMV, among others). The coding portion of the genome is preceded and followed by relatively short untranslated regions (UTRs) of 147 nt (5′UTR) and 251 nt (3′UTR).

A large, 3095-codon-long ORF covering approximately 96% of the whole genome codes for a polyprotein with an estimated *Mr* of 353.8 kDa (~354 K). In silico analyses suggested the polyprotein processing at the cleavage sites presented in Figure 4. Similar to other potyviruses, this 354K polyprotein is presumably cleaved by three virus-encoded proteases to give ten mature products (from N- to C-terminus): P1 (the first protein), HC-Pro (helper component proteinase), P3, 6K1 (the first 6 kDa protein), CI (cylindrical inclusion protein), 6K2 (the second 6 kDa protein), NIa-VPg (viral genome-linked protein), NIa-Pro (nuclear inclusion body a-proteinase), NIb (nuclear inclusion body b), and CP (coat protein) [11]. Analyses of the amino acid sequences showed the presence of conserved motifs reported in orthologs of other potyviruses. The three amino acid motifs reported to have a function in the potyvirus aphid-mediated virus transmission have been identified in the HC-Pro (PTK and KITC) and CP proteins (DAG). A short ORF, referred to as PIPO (Pretty Interesting Potyviridae ORF) [29], which involved viral intercellular movement [30], was found overlapping the P3 coding region of the large ORF (nt positions 2931–3158).

In the initial BLAST alignments performed on complete nucleotide sequences in July 2020, KudCRBV shared the greatest level of identities (87% and 94%, respectively) with the two near-complete sequences deposited in GenBank as wisteria vein mosaic virus isolates Ce-JH (WVMV-Ce-JH; acc. no. LC729727.1) and JEBUp (WVMV-JEBUp; acc. no. MT603851.1). However, identity with a complete sequence of an original isolate of WVMV from Beijing (WVMV-Beijing; acc. no. AY656816.1) [31] was found to be only 76.2%—a value too close to the currently applied species demarcation criteria (<76% nt identity). Similar values were obtained in comparisons with an Iranian WVMV isolate with complete sequences available in GenBank (WVMV-Ir; acc. no. MN514947.1) [32].

**Figure 5:**
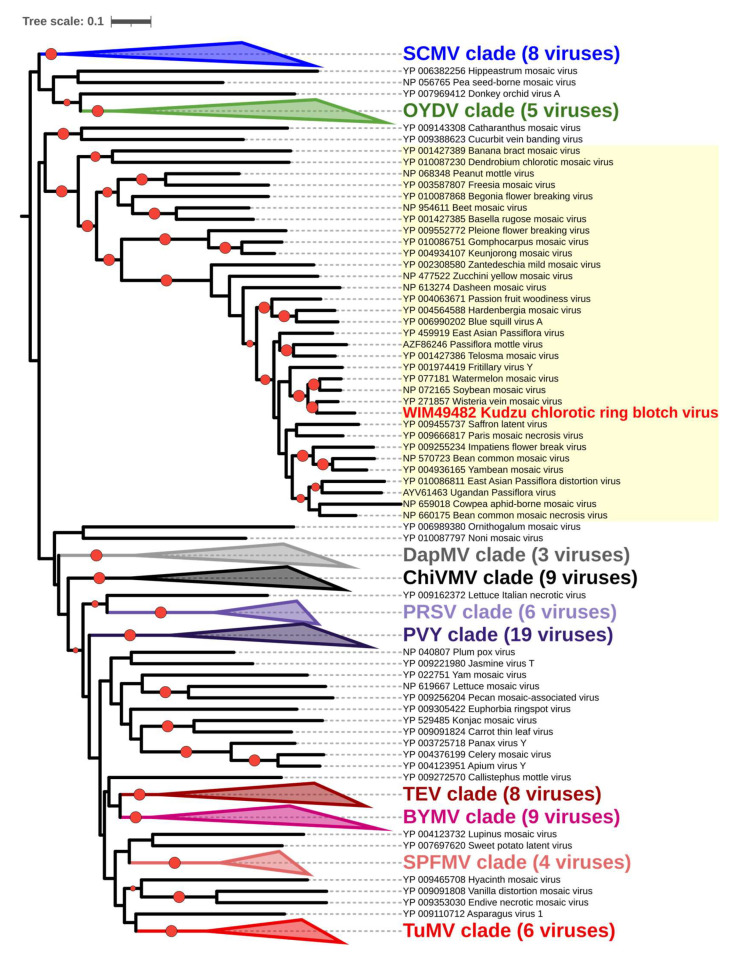
Phylogenetic tree showing relationships of kudzu chlorotic ring blotch virus (KudCRBV) to members of the genus *Potyvirus*. The tree was generated by IQ-tree [24] on TrimAl [22] curated amino acid sequence alignment of the entire polyproteins encoded by KudCRBV and members of 135 species in the genus *Potyvirus*. Visual presentation of the tree was achieved with iTOL [26]. Red circles indicate clades with >95% support. Accession numbers of sequences used in the analysis are indicated adjacent to the virus names. For improved visualization, several well-supported clades were collapsed. Abbreviations for “type members” of those clades include bean common mosaic virus (BCMV), bean yellow mosaic virus (BYMV), chili veinal mottle virus (ChiVMV), daphne mosaic virus (DapMV), onion yellow dwarf virus (OYDV), papaya ringspot virus (PRSV), potato virus Y (PVY), sugarcane mosaic virus (SCMV), sweet potato feathery mottle virus (SPFMV), tobacco etch virus (TEV), and turnip mosaic virus (TuMV). Yellow shading indicates the “bean common virus clade” with the inclusion of KudCRBV.

In phylogenetic analyses performed on complete amino acid sequences of the polyproteins of reference potyviral isolates, the virus from kudzu was placed in a well-supported subclade composed of soybean mosaic virus (SMV), watermelon mosaic virus (WMV), and wisteria vein mosaic virus (WVMV) within the “bean common mosaic virus clade” (“BCMV clade”, synonym: “BCMV group”) of potyviruses (Figure 5). Indeed, KudCRBV-Ack01 represents a sister branch to WVMV (Beijing isolate), with the mutual evolutionary distances comparable to those between SMV and WMV (Figure 5).

### 3.3. Genome Sequence Analyses of the Three Isolates of Wisteria Vein Mosaic Virus from Mississippi

Thus, inconclusive results from initial comparisons of KudCRBV with a limited number of the WVMV genome sequences available at the time of the initial analyses (July 2020) regarding their relationships, as well as overall taxonomic position of a kudzu virus within the genus *Potyvirus*, prompted a state-wide survey of wisterias in MS that aimed to identify suitable sources and to generate molecular data on local isolates of this virus. As a result of that survey effort, three wisteria samples were determined to be positive for WVMV following potyvirus-specific DAS-ELISA. The resulting virus-positive plant material was submitted to HTS sequencing by Illumina.

After raw sequence assembly and the performance of 5′RACE experiments for the first two isolates, their complete genome sizes differed by a single nucleotide (not counting the polyA tail): 9694 vs. 9693 nt for isolates MS14-19 and MS20-26. Additionally, a coding-complete sequence 9664 nt in size containing partial 5′UTR and entire coding and 3′UTR sequences was assembled only from HTS reads for the isolate MS12-11. Genomes of the three isolates coded for a major polyprotein with a molecular mass of ~354 kDa and a small PIPO protein 74 aa in size.

Pairwise comparisons revealed that the WVMV population present in MS is rather conserved, as the genomes of the three isolates shared mutual nt identities ranging from 95.3% to 98.9%, depending upon the specific combination used for comparison. Putative polyproteins of the three WVMV isolates shared identical lengths of 3092 amino acids and had highly conserved contents, differing mutually by only 1% (MS14-19 vs. MS20-26) to 2.7% (MS14-19 vs. MS12-11). However, they shared only 76.4–76.6% nt sequences genome-wide with KudCRBV.

### 3.4. Comparison of KudCRBV with Allied Viruses

Generation of data on genomes of the three local WVMV isolates, along with timely availability of sequences of two German sources of this virus from the DSMZ collection, allowed for a more robust analysis to understand the nature of KudCRBV-Ack01 relationships to allied viruses (see Table S2 for data on WVMV isolates).

**Figure 6:**
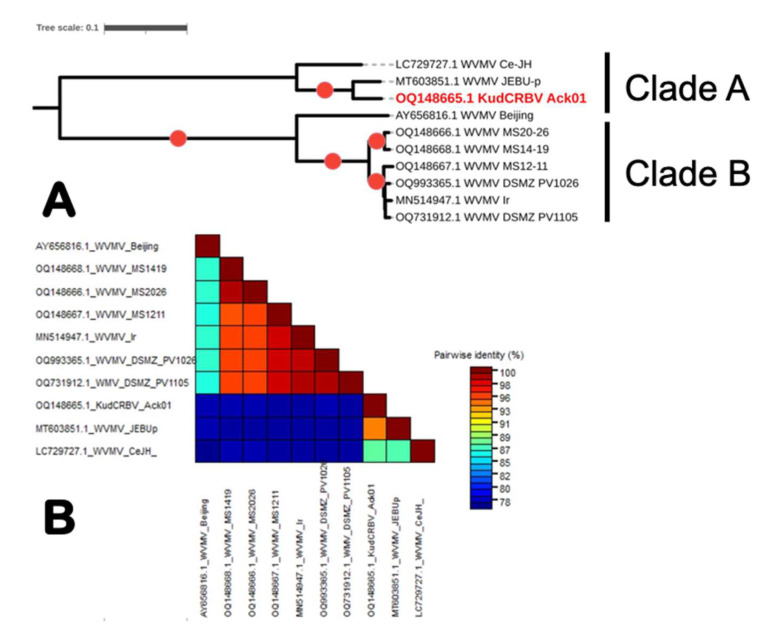
Relationship of kudzu chlorotic ring blotch virus Ack01 (KudCRBV-Ack01) and isolates of wisteria vein mosaic virus (WVMV) with available complete or near-complete genome sequences in GenBank. (**A**) Mid-point-rooted maximum-likelihood tree showing relationships of KudCRBV-Ack01 with several viruses with genomic sequences deposited in GenBank as isolates of wisteria mosaic virus (WVMV). The tree was generated by IQ-tree [24] on the MAFFT-generated alignment [21] of complete nucleotide sequences under the best-fit substitution model, GTR + F + I, and visualized with iTOL [26]. Red circles indicate clades with >95% support. Clear-cut separation into two distinct groups with genetic distances between clades approximating species demarcation criteria in the genus *Potyvirus*. (**B**) Color-based representation of pairwise identities between KudCRBV-Ack01 and WVMV isolates. Analyses were performed with the SDT v 1.2 program [20].

Phylogenetic analyses of either complete ORF (nt) or polyprotein (aa) sequences revealed the presence of two distinct clusters, one represented by KudCRBV and two WVMV isolates (Ce-JH and JEBUp) reported from non-wisteria hosts from Korea (“Clade A”; Figure 6A) and the other containing all isolates from *Wisteria sinensis* independent of geographic origin, including the “type isolate” WVMV-Beijing and three isolates from MS, along with those of Iranian and German provenance (“Clade B”). Similar tree topologies were obtained with a richer dataset which included a few additional isolates from Italy, Iran, Australia, and two from China; KudCRBV and two WVMV isolates from Korea formed an evolutionarily distinct lineage from the Wisteria-infecting WVMV isolates.

Direct comparison of KudCRBV genome sequences showed a limited level of identity with WVMV isolates from “Clade A”, ranging from 76.2% to 76.5%, while they were more related to isolates from Korea (87% and 94% nt identity). KudCRBV was found to be most related to WVMV-JEBUp, an isolate reported from soybean [33].

On the other hand, three WVMV isolates from MS were closely related to two recently reported German isolates, WVMV-PV1026 (GenBank OQ993365.1) and WVMV-PV1105 (GenBank OQ731912.1), with no currently associated publication, and an Iranian isolate WVMV-Ir (GenBank MN514947.1) [32] sharing 95.4–98.3% identical nt. Importantly, all three isolates shared high levels of identity spanning from 88.5% to 89.5% with a partial sequence (1007 nt) of a WVMV isolate from the state of Washington [34].

To better understand how the genetic distances observed in analyses of KudCRBV and WVMV populations compare to those between populations of WMV and SMV, we included several isolates of those two viruses in the next round of analyses. KudCRBV shared similar levels of identity, around 73–74%, with SMV and WMV isolates. The highest level of mutual sequence identities was observed between SMV and WMV, varying between 78.8% and 79.2%, therefore significantly exceeding that between KudCRBV and WVMV isolates from wisteria (Figure S1 and Table S3). Similarly, identities of aa sequences were always lower in the case of KudCRBV vs. WVMV compared to SMV vs. WMV isolates (Figure S1 and Table S3).

The identity levels of ten KudCRBV mature proteins with those encoded by closely related viruses, deduced by in silico processing of the primary polyproteins, varied according to the protein and virus used for comparison (Table S4). Of importance is to notice that in the case of taxonomically relevant coat protein (CP) sequences, KudCRBV seems to be equidistant (80–81%) from the counterparts encoded by the WVMV isolates from “Clade B” or by the SMV or WMV sequences used in the analyses.

### 3.5. Survey and Transmission Experiments

Once the viral cause of the symptoms on the kudzu contained in the patch in Ackermann, MS, was identified, two KudCRBV-centered surveys were conducted. In the first survey, which aimed to investigate possible additional natural hosts of the virus, foliar tissue was collected from 66 plants belonging to nine different botanical species from the area immediately surrounding the infected kudzu and tested by RT-PCR for the virus. No plant sample other than kudzu was determined to be infected by KudCRBV.

**Figure 7:**
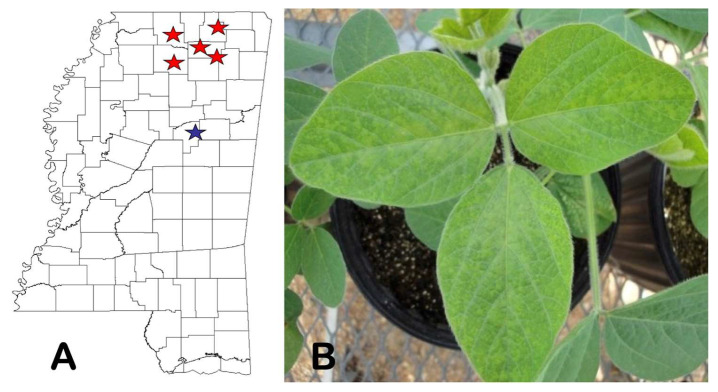
(**A**) Map of Mississippi with approximate locations of kudzu chlorotic ring blotch virus (KudCRBV)-infected kudzu patches. A blue star represents the original source of the virus, while the red ones indicate additional infections revealed during the survey. (**B**) Mild chlorosis and systemic mosaic/vein discoloration symptoms on the trifoliate of a soybean plant mechanically inoculated with KudCRBV-Ack01.

A state-wide survey of 103 kudzu samples collected from various locations revealed 5 positive samples in DAS-ELISA with “universal” potyvirus monoclonal antibodies. Additional RT-PCR tests, followed by sequencing of 349 bp long amplicons confirmed that all five samples were indeed infected by KudCRBV, as they shared high levels (97–98.5%) of identical nucleotide sequences. Curiously, all six (the original and five newly discovered) kudzu patches were in the northernmost part of the state (Figure 7A).

Vector transmission trials were successful, although at variable rates. The cotton aphid transmitted the virus to soybean (two out of five plants) and kudzu accession no. 9227 (a total of 2/5), while no infections developed in kudzu no. 434246. On the other hand, the potato aphid successfully inoculated both kudzu genotypes, but did not transfer KudCRBV to any of the five soybean plants of the AG4903 cultivar used in these trials. The RT-PCR experiments showed transmission to two out of five plants of kudzu no. 9227 and only to a single plant in the case of kudzu no. 434246.

In mechanical transmission attempts, KudCRBV caused systemic infections in 5 out of 20 soybean plants and 4 out of 12 cv ‘Bountiful’ bean plants. KudCRBV provoked systemic veinal discoloration/clearing, chlorosis, and mosaic in AG4903 soybean plants approximately two weeks post-inoculation (Figure 7B). In French beans, the virus induced a symptomatologic response like the one in soybean—a systemic infection manifested as mild chlorotic mosaic. No other plants used in these experiments were susceptible to KudCRBV, as ascertained by the DAS-ELISA test, performed at 30 d.p.i., and RT-PCR, performed a week after. However, repeated attempts to possibly identify KudCRBV infections in soybean plants with clear virus-like symptoms by testing a total of 85 samples collected from a few commercial soybean fields located up to ~5 km from the original kudzu patch in Ackerman did not yield any positive result.

## 4. Discussion and Conclusions

The virus discovered and characterized in the current report is herein described as a new member of the genus *Potyvirus*, for which the name kudzu chlorotic ring blotch virus (KudCRBV), based on the natural host and the originally observed symptoms, is proposed. The virus shares properties with previously characterized potyviruses, as it induced cytoplasmic structures within infected kudzu plant material that are typical of infections by this group of viruses and is aphid transmissible. Indeed, the virus has been successfully transmitted by the two aphid species used in the study under experimental conditions. Taking into consideration the relatively widespread occurrence of cotton and melon aphids in MS, we cannot help but suggest their role in the epidemiology of the virus in nature. Although the survey performed on plant samples belonging to nine different botanical species collected from the vicinity of the original kudzu patch in Ackerman, MS, USA, failed to identify additional natural hosts of this virus, transmission trials performed under controlled conditions also determined that KudCRBV can infect two additional leguminous hosts. Of particular significance is the fact that soybean, one of the major agricultural commodities in the state, was determined to be susceptible to KudCRBV infection using both an inoculation procedure with cotton aphids and by mechanically rubbing virus-infested sap onto soybean leaflets. Of additional noteworthy importance is the fact that kudzu has been reported to occur in 81 of the 82 counties in Mississippi [35]. Many of the counties that contain kudzu also grow a substantial number of soybean hectares, with soybean produced in approximately 76 counties on an annual basis.

In addition to the original kudzu patch in Ackerman, the state-wide survey revealed several additional KudCRBV foci in the state. The five additionally infected kudzu plants were all collected in the northern part of the state (five infections in 49 samples collected north of Ackermann), while KudCRBV was not detected in any of the 54 samples collected from the central or southern part of Mississippi. It is not clear whether this distribution pattern is a result of the use of KudCRBV-infected material during a major planting campaign almost a century ago or of natural spread over time either by airborne vectors (aphids) or possibly via seed-transmission, with the possible involvement of birds as major vehicles for their transport over greater distances. Moreover, given that kudzu seed requires a level of scarification prior to germination, it is more likely that the movement of virus-infested seed by birds has occurred. However, further studies of possible pollen and/or seed transmission might shed light on the current virus distribution in Mississippi. It is likely that there are more virus-infected kudzu patches in Mississippi, since the invasive weed is very widespread throughout the state and only a fraction of its population has been sampled and tested in this work.

Generally, the discovery of pathogenic viruses in an invasive and destructive plant such as kudzu might appear to be an exciting opportunity for their employment as part of an integrated control strategy. However, the use of exotic viruses, such as KudCRBV, as biological control agents could be highly controversial as it poses a risk for economically important crops.

Although we have no experimental proof of natural infections of soybean by KudCRBV in MS at this time, our results clearly demonstrated the susceptibility of this crop to the virus. This fact should not be overlooked considering the economic importance of soybean production in MS and more broadly in the U.S. and possibly warrants an increased awareness regarding the potential future threat to soybean. There are several well-studied cases of virus “spillover” from their natural non-cultivated hosts (wild reservoirs) and adaptation to agricultural ecosystems/crops worldwide (for a review, see [36]). It should not be forgotten that soybean is one of the closest botanical relatives of kudzu in the Southern U.S., as they both belong to the Fabaceae, and that KudCRBV is closely related to SMV, a potyvirus known to be a major problem for soybean production worldwide [37]. Furthermore, upon completion of our transmission experiments, an independent report on an unusual WVMV isolate from a study of the soybean virome in South Korea was published [33], indirectly corroborating our experimental results. Finally, KudCRBV was found to be the closest relative of the WVMV isolate from soybean (94.1% nt identity).

According to currently valid molecular demarcation criteria, members of different species in the genus *Potyvirus* have genome sequences that are generally less than 76% identical in nt content, and their encoded polyproteins share less than 82% identical amino acids. The thresholds for species demarcation using nucleotide identity values for the individual coding regions range from <58% for P1 up to <80% aa identity for CP [11,38,39]. However, additional criteria for recognition of new species may include original host and other biological features.

KudCRBV likely originated in the Far East and was introduced in the U.S. via infected planting material during in the late 19th or early 20th century. Indeed, it is evolutionarily most closely related to WVMV and a few additional viruses within the “SMV cluster” of the “BCMV clade”, one of two of the largest lineages of potyviruses that apparently radiated in Southeast and East Asia approximately 3600 years before present (ybp) [40,41,42]. Of particular interest for the current study is a sublineage composed of three currently recognized viruses infecting hosts belonging to the families Fabaceae (SMV and WVMV) and Cucurbitaceae (WMV) to which KudCRBV is most closely related.

WVMV is a member of the genus *Potyvirus*, of Asian origin, known to affect plants belonging to several species in the genus *Wisteria* (*Wisteria sinensis*, *W. floribunda*, *W. brachybotrys*, and *W. venusta*) causing Wisteria mosaic disease, a disease originally reported from the U.S. in the mid-20th century [43]. However, the viral origin of the disease was demonstrated for the first time in 1970 in a study from the Netherlands by the observation of filamentous virions reminiscent of potyviruses in infected tissue [44]. The virus was reported from *Wisteria* spp. in various places worldwide, including different European countries, including the United Kingdom [45], Poland [46], and, most recently, Italy [47]. The virus has also been reported from the same host in the U.S. [34], New Zealand [48], and on multiple occasions in Iran [32,49,50], as well as various provinces of China [31,51,52]. With a few exceptions, these records relied upon the detection of the virus via RT-PCR and sequencing corresponding amplicons, so that the genomes of only four isolates were completely (or near-completely) sequenced. To further corroborate our data, we identified and sequenced three local WVMV isolates from *W. sinensis* collected in MS. Fortuitously, the genome data of two German isolates of the virus from the DSMZ collection were made publicly available just in time for the preparation of this manuscript and were included in the analyses.

Phylogenetic analyses and pairwise analyses performed in the current work, independent of the type of dataset used (complete genome sequences, polyprotein sequences, or coat protein sequences), revealed a clear-cut divergence of known WVMV populations into two distinct lineages (Clades A and B; Figure 6), one of which included KudCRBV. Molecular differences between members of the two lineages approximated those recommended for species demarcation by the ICTV *Potyviridae* Study Group (SG) [11], suggesting that some of the WVMV sequences may need reclassification and possibly reannotation. A similar call for reannotation was recently made for the SMV isolate Uraria (GenBank LC037232.1) characterized from *Uraria crinita* in 2014, which was found to be significantly different from the sequences of another 304 isolates used in that comprehensive study [53] and sufficiently distinct to be considered representative of a new potyviral species.

While the identities of KudCRBV with two genomes deposited as isolates of WVMV significantly exceed the proposed species demarcation criteria for the genus *Potyvirus*, suggesting that these three viruses belong to the same taxon, it is questionable whether they should be grouped together with the rest of the WVMV isolates, as they clearly belong to an evolutionary distinct lineage. In phylogenetic trees, distances between the two WVMV clades are comparable to that between SMV and WMV, which are classified as two different species (Figure S2). Moreover, identity values between the genomes of SMV and WMV isolates and their major expression products exceed the recommended thresholds for a few percentages and are always slightly greater than those between KudCRBV, WVMV-JEBUp, and WVMV-Ce-JH versus an additional seven isolates of WVMV (Tables S3 and S4). Further analyses involving a comparison of amino acid sequences surrounding putative cleavage sites showed clear-cut differences between isolates belonging to Clades A and B in five of nine sites. Interestingly, another four cleavage sites were universally conserved not only in WVMV isolates, but also in several isolates of WMV and SMV. On the other side, WMV and SMV isolates differed in amino acid sequences of only three out of nine cleavage sites between them. Again, these results suggest KudCRBV classification in a new taxon within the genus *Potyvirus*.

The three WVMV isolates from wisteria collected from MS were grouped with the other isolates from the same host (*W. sinensis*) independently of their geographic origin (Clade B; Figure 6A). The clade included the originally sequenced (“type”) isolate from China [31], an isolate characterized from Iran [32], and two German isolates (no associated publication), along with the three isolates from MS. On the other hand, the three virus isolates forming a sister clade (“Clade A”; Figure 6A) are reported from additional hosts; KudCRBV-Ack01 was isolated from kudzu (*Pueraria montana* var. *lobata*) collected in the Southern U.S., whereas the other two were characterized from two distinct hosts in Korea, WVMV-Ce-JH from jack bean (*Canavalia ensiformis*) and WVMV-JEBUp from soybean. Therefore, this group of viruses have a rather distinct gamma of hosts compared to those from “Clade B” (Figure 6A).

In conclusion, KudCRBV represents a borderline case from the taxonomic standpoint and its position among other potyviruses may be argued both ways: it could be representative of a new species (embracing two closely related virus isolates, JEBUp and Ce-JH) or it could be “lumped” together with all the WVMV isolates as a distinct strain adapted to a particular host (kudzu). Based on the extensive results from this study, in particular an in-depth comparison of genetic divergences between populations of closely related viruses (i.e., SMV vs. WMV), which are classified as different species, our opinion is that the process of speciation in the case of KudCRBV (and the other two WVMV isolates) has been sufficiently completed to warrant the creation of a new and distinct taxon in the genus *Potyvirus* to classify these viruses. However, we leave the final decision regarding the taxonomic allocation of the virus from kudzu to the experts from the ICTV *Potyviridae* Study Group.

## Data Availability

Complete genome sequences of KudCRBV (acc. no. OQ148665) and three isolates of WVMV (acc. no. OQ148666-OQ148668) were deposited in GenBank.

## Supplementary Materials

The following supporting information can be downloaded at: https://www.mdpi.com/article/10.3390/v15112145/s1, Figure S1: Color-based representation of pairwise identities between KudCRBV-Ack01 and isolates of the three most closely related viruses (WVMV, WMV, and SMV). Analysis was performed with the SDT v 1.2 program, Figure S2: Maximum-likelihood tree showing relationships of KudCRBV-Ack01 with viruses from the “SMV subclade” of potyviruses, Figure S3: CP-based ML tree showing relationships of KudCRBV and WVMV isolates, Table S1: List of plants collected from the vicinity of the kudzu patch infected with kudzu chlorotic ring blotch virus (KudCRBV) in Ackerman, MS, USA, and tested in DAS-ELISA, Table S2: General information on isolates of wisteria vein mosaic virus (WVMV) used for comparisons with kudzu chlorotic ring blotch virus (KudCRBV), Table S3: Percentages of nucleotide identities of kudzu chlorotic ring blotch virus (KudCRBV-Ack01) and isolates of wisteria vein mosaic virus (WVMV), watermelon mosaic virus (WMV), and soybean mosaic virus (SMV), Table S4: Percentages of amino acid identity of the ten mature proteins encoded by the large ORF of kudzu chlorotic ring blotch virus, isolate Ack01, with the most closely related viruses.
