# Supplementary material for: Characterization of a Putative New Member of the Genus Potyvirus from Kudzu (Pueraria montana var. lobata) in Mississippi
Source: Viruses. 2023 Oct 25;15(11):2145. doi: 10.3390/v15112145 (PMC10675740; doi:10.3390/v15112145)

Aboughanem-Sabanadzovic et al,

Characterization of a new member of the genus *Potyvirus* from kudzu (*Pueraria montana* var. *lobata*) in Mississippi

Table S1. List of plants collected from the vicinity of kudzu patch infected with kudzu chlorotic ring blotch virus (KudCRBV) in Ackerman, MS and resulted negative in DAS-ELISA.

Common plant name	Botanical species name	Number of plants tested
Trumpet creeper	*Campsis radicans*	12
Chinese privet	*Ligustrum sinense*	2
Carolina horsenettle	*Solanum carolinense*	8
Narrowleaf plantain	*Plantago lanceolata*	6
Purpletop vervain	*Verbena bonariensis*	5
Ryegrass	*Lolium* sp	5
Silverleaf	*Leucophyllum frutescens*	6
Goldenrod	*Solidago* sp	10
Clover	*Trifolium* spp	12

Table S2. General information on isolates of wisteria vein mosaic virus (WVMV) used for comparisons with kudzu chlorotic ring blotch virus (KudCRBV).

Sequence Acc No	Isolate name	Host	Country	Genome size
LC729727.1	Ce-JH	*Canavalia ensiformis*	South Korea	9674
MT603851.1	JEBUp	*Glycine max*	South Korea	9668
MN514947.1	Ir	*Wisteria sinensis*	Iran	9705
AY656816.1	Beijing	*Wisteria sinensis*	China	9695
OQ993365.1	PV-1026	*Wisteria sinensis*	Germany	9694
OQ731912.1	PV-1105	*Wisteria sinensis*	Germany	9693
OQ148668.1	MS14-19	*Wisteria sinensis*	USA	9694
OQ148667.1	MS12-11	*Wisteria sinensis*	USA	9664
OQ148666.1	MS20-26	*Wisteria sinensis*	USA	9693

Table S3. Percentages of nucleotide identities of kudzu chlorotic ring blotch virus (KudCRBV-Ack01) and isolates of wisteria vein mosaic virus (WVMV), watermelon mosaic virus (WMV) and soybean mosaic virus (SMV).

1: OQ148665.1_KudCRBV	100.00	94.07	87.25	**76.29**	**76.66**	**76.53**	**76.42**	**76.49**	**76.45**	**76.51**	74.29	73.90	73.73	73.85	73.51	73.30	73.38	73.14	73.27	73.25	73.15	73.01
2: MT603851.1_WVMV_JEBUp	94.07	100.00	87.25	**76.44**	**76.49**	**76.49**	**76.54**	**76.55**	**76.50**	**76.53**	73.95	73.70	73.51	73.37	73.50	73.23	73.13	73.06	73.21	73.17	73.22	72.95
3: LC729727.1_WVMV_Ce_JH	87.25	87.25	100.00	**75.89**	**76.70**	**76.64**	**76.49**	**76.42**	**76.38**	**76.40**	73.87	73.83	73.54	73.66	73.05	73.15	72.95	72.93	73.02	73.05	72.95	72.83
4: NC_007216.1_WVMV_Beijing	76.29	76.44	75.89	100.00	85.95	85.86	85.57	85.81	85.71	85.94	74.02	74.18	73.80	74.11	74.23	73.91	74.15	74.41	74.23	74.26	74.32	73.81
5: OQ148666.1_WVMV_MS20_26	76.66	76.49	76.70	85.95	100.00	98.93	95.36	95.68	95.64	95.51	74.56	74.27	74.36	74.47	74.40	74.15	74.40	74.44	74.20	74.24	74.08	74.07
6: OQ148668.1_WVMV_MS14_19	76.53	76.49	76.64	85.86	98.93	100.00	95.33	95.61	95.58	95.44	74.47	74.16	74.42	74.38	74.32	74.10	74.27	74.31	74.17	74.20	74.03	73.96
7: OQ148667.1_WVMV_MS12_11	76.42	76.54	76.49	85.57	95.36	95.33	100.00	98.27	98.28	98.17	74.59	74.13	74.41	74.22	74.04	73.89	74.14	74.03	73.80	73.88	73.86	73.87
8: OQ993365.1_WVMV_PV1026	76.49	76.55	76.42	85.81	95.68	95.61	98.27	100.00	98.92	98.69	74.36	74.08	74.31	74.25	74.15	74.10	74.30	74.15	73.90	73.98	73.97	73.97
9: OQ731912.1_WVMV_PV1105	76.45	76.50	76.38	85.71	95.64	95.58	98.28	98.92	100.00	98.80	74.35	74.02	74.28	74.18	74.10	73.97	74.21	74.07	73.88	73.96	73.95	73.91
10: MN514947.1_WVMV_Ir	76.51	76.53	76.40	85.94	95.51	95.44	98.17	98.69	98.80	100.00	74.43	74.08	74.33	74.14	74.09	74.01	74.24	74.07	73.88	73.98	73.91	73.85
11: OQ161637.1_SMV_Cheorwon	74.29	73.95	73.87	74.02	74.56	74.47	74.59	74.36	74.35	74.43	100.00	96.75	96.16	93.95	78.94	78.91	79.08	78.92	78.56	78.64	78.45	78.23
12: FJ640980.1_SMV_G6	73.90	73.70	73.83	74.18	74.27	74.16	74.13	74.08	74.02	74.08	96.75	100.00	95.85	93.78	79.16	79.12	79.21	79.01	78.67	78.71	78.58	78.51
13: AY216010.1_SMV_G7	73.73	73.51	73.54	73.80	74.36	74.42	74.41	74.31	74.28	74.33	96.16	95.85	100.00	93.67	78.85	78.78	78.91	78.82	78.49	78.55	78.47	78.15
14: NC_002634.1_SMV_N	73.85	73.37	73.66	74.11	74.47	74.38	74.22	74.25	74.18	74.14	93.95	93.78	93.67	100.00	78.84	78.73	78.75	78.79	78.27	78.37	78.33	78.03
15: KM597070.1_WMV_RKG	73.51	73.50	73.05	74.23	74.40	74.32	74.04	74.15	74.10	74.09	**78.94**	**79.16**	**78.85**	**78.84**	100.00	92.51	93.20	93.04	92.77	92.87	92.50	92.17
16: MN854643.1_WMV_GC-wm2	73.30	73.23	73.15	73.91	74.15	74.10	73.89	74.10	73.97	74.01	**78.91**	**79.12**	**78.78**	**78.73**	92.51	100.00	94.44	94.24	90.82	90.85	90.39	90.07
17: OQ136662.1_WMV_JY16	73.38	73.13	72.95	74.15	74.40	74.27	74.14	74.30	74.21	74.24	**79.08**	**79.21**	**78.91**	**78.75**	93.20	94.44	100.00	93.93	90.99	91.04	90.40	90.43
18: KT992090.1_WMV_Yeongju73	73.14	73.06	72.93	74.41	74.44	74.31	74.03	74.15	74.07	74.07	**78.92**	**79.01**	**78.82**	**78.79**	93.04	94.24	93.93	100.00	90.86	90.90	90.64	90.45
19: JF273462.1_WMV_C06_526	73.27	73.21	73.02	74.23	74.20	74.17	73.80	73.90	73.88	73.88	**78.56**	**78.67**	**78.49**	**78.27**	92.77	90.82	90.99	90.86	100.00	99.44	97.72	93.84
20: EU660578.1_WMV_FMF00LL2	73.25	73.17	73.05	74.26	74.24	74.20	73.88	73.98	73.96	73.98	**78.64**	**78.71**	**78.55**	**78.37**	92.87	90.85	91.04	90.90	99.44	100.00	97.83	93.93
21: OQ847407.1_WMV_PV-1047	73.15	73.22	72.95	74.32	74.08	74.03	73.86	73.97	73.95	73.91	**78.45**	**78.58**	**78.47**	**78.33**	92.50	90.39	90.40	90.64	97.72	97.83	100.00	93.68
22: NC_006262.1_WMV_Fr	73.01	72.95	72.83	73.81	74.07	73.96	73.87	73.97	73.91	73.85	**78.23**	**78.51**	**78.15**	**78.03**	92.17	90.07	90.43	90.45	93.84	93.93	93.68	100.00

Red bold font indicates percentages of genome-wide nt identities between KudCRBV, WVMV-JEBUp and WVMV-Ce-JH with WVMV isolates.

Yellow highlighted are percentages of genome-wide nt identities between isolates of SMV and WMV.

Table S4. Percentages of amino acid identity of the ten mature proteins encoded by the large ORF of kudzu chlorotic ring blotch virus, isolate Ack01, with most closely related viruses.

	P1	HC-PRO	P3	6K1	CI	6K2	VPg	NIa-Pro	NIb	CP
WVMV Clade A[1]	84-91	98-99	97-99	96-98	99	94-98	97-99	97-98	97-99	95-98
WVMV Clade B[2]	47-48	87-88	70-72	86-88	93	91-92	91	90-91	87-88	80-81
SMV[3]	45-48	86-88	64-66	83-85	91-92	77-78	88-89	84-86	84-85	80-81
WMV[4]	44-46	84	65-66	85-87	90-91	81-83	87-88	86-87	85-86	78-80

Legend:

[1] -average identity values with wisteria vein mosaic virus isolates JEBU-p and Ce-JH (GenBank Accessions MT603851.1 and LC729727.1, respectively)

[2] - average identity values with wisteria vein mosaic virus isolates Beijing, Iran, MS20-26, MS12-11, MS14-19, PV1026 and PV1105 (GenBank Accessions AY656816.1; MN514947.1; OQ148666.1; OQ148667.1; OQ148668.1; OQ993365.1 and OQ731912.1, respectively)

[3] -average identity values with 15 most closely related sequences of soybean mosaic virus (SMV)

[4] -average identity values with 15 most closely related sequences of watermelon mosaic virus (WMV)

Figure S1. Color-based representation of pairwise identities between KudCRBV-Ack01 and isolates of the three most closely related viruses (WVMV, WMV and SMV). Analyses were performed with SDT v 1.2 program [Muhire et al, 2014].

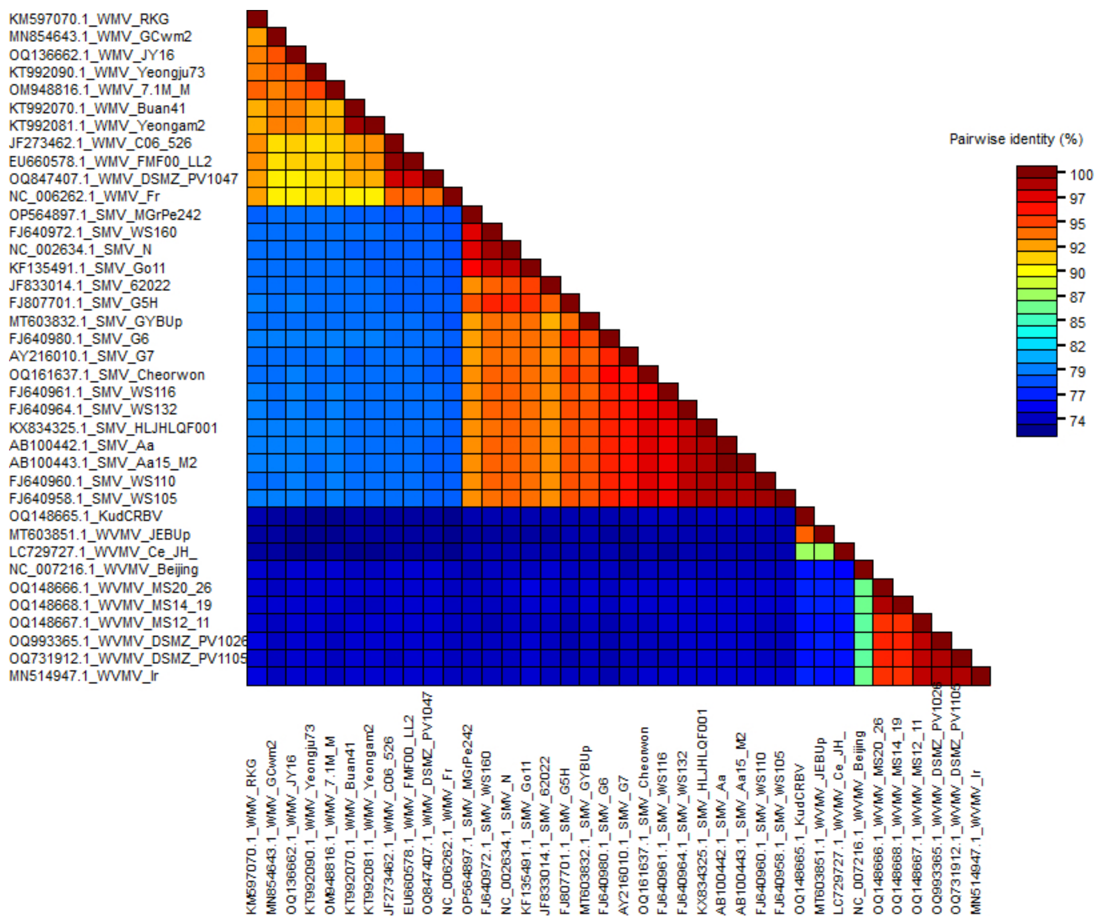

Figure S2. Maximum likelihood tree showing relationships of KudCRBV-Ack01 with several viruses with genomic sequences deposited in the GenBank as isolates of wisteria mosaic virus (WVMV) and a few isolates of soybean mosaic virus (SMV and watermelon mosaic virus (WMV). Tree was generated by IQtree on the MAFFT-generated alignment [Katoh and Standley, 2013] of complete nucleotide sequences under the best-fit substitution model GTR+F+I and visualized with the iTOL [Letunic and Bork, 2022]. Different color shades indicate different lineages, corresponding to possibly four distinct species.

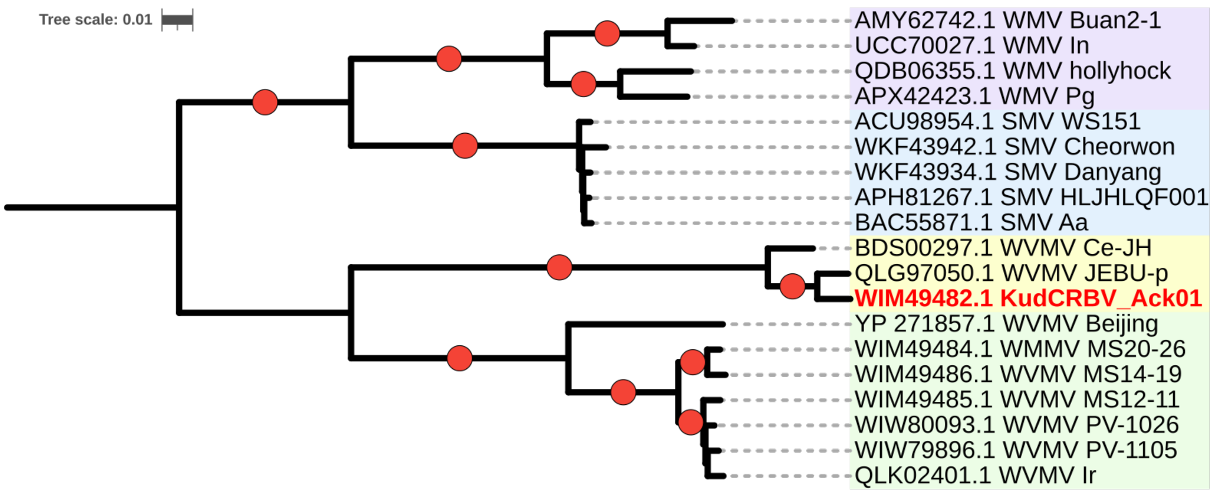

Figure S3. Maximum likelihood tree showing relationships of KudCRBV-Ack01 with several viruses with genomic sequences deposited in the GenBank as isolates of wisteria mosaic virus (WVMV) and a few isolates of soybean mosaic virus (SMV and watermelon mosaic virus (WMV). Tree was generated by IQtree on the MAFFT-generated alignment [Katoh and Standley, 2013] of coat protein gene under the best-fit substitution model TN+F+I and visualized with the iTOL [Letunic and Bork, 2022]. Different color shades indicate different lineages, corresponding to possibly four distinct species. Input data 15 sequences with 846 nt sites of which invariant were 576 (68%).

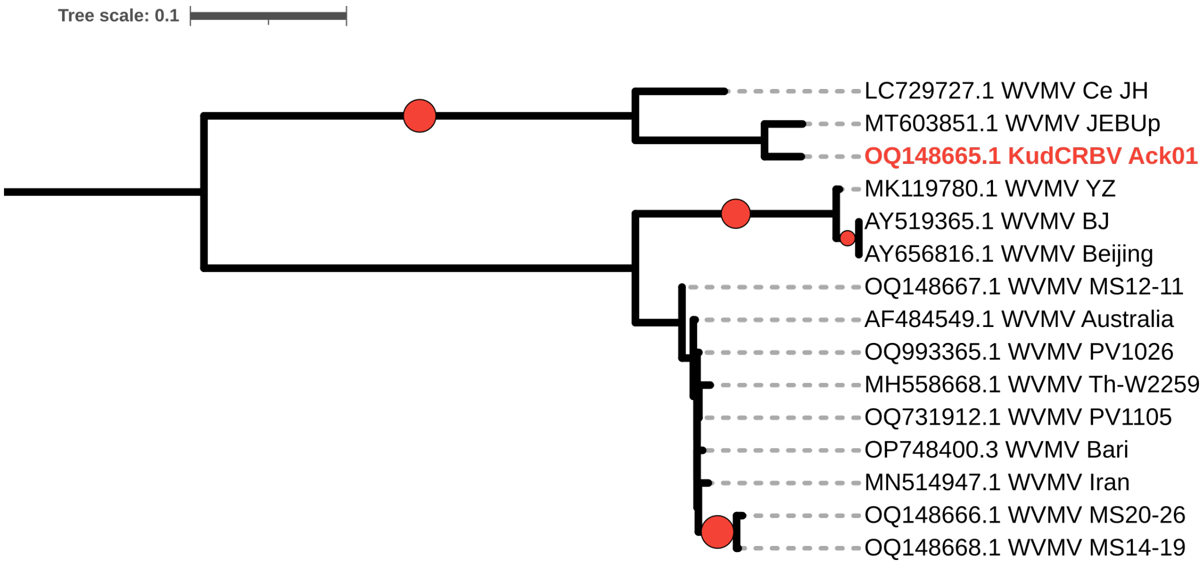

Supplement: Supplementary file 1 [file viruses-15-02145-s001.zip › viruses-2648299-supplementary.pdf]
